# Female genital schistosomiasis in Ghana: An exploration of knowledge, attitudes, and practice among women of reproductive age

**DOI:** 10.1016/j.puhip.2025.100632

**Published:** 2025-06-26

**Authors:** Comfort D. Tetteh, Alfred K. Manyeh, Jabulani R. Ncayiyana, Themba G. Ginindza

**Affiliations:** a School of Nursing and Public Health, College of Health Sciences, University of KwaZulu-Natal, Durban, South Africa; b Ghana Health Service, Ayawaso East Municipal Health Directorate, Nima, Accra, Greater Accra Region, Ghana; c Intitute of Health Research, The University of Health and Allied Sciences, Ho, Volta Region, Ghana; d Cancer & Infectious Disease Epidemiology Research Unit (CIDERU), College of Health Sciences, University of KwaZulu-Natal, South Africa

**Keywords:** Female genital schistosomiasis, Knowledge, Attitudes, Practices, Women of reproductive age, Ghana

## Abstract

**Objective:**

Female genital schistosomiasis (FGS) affects about 11 % of women of reproductive age in Ghana. The disease remains insignificant and poorly understood in endemic communities and healthcare professionals across Sub-Saharan Africa. The objective was to assess the knowledge, attitudes, and practices among women of reproductive age towards FGS.

**Study design:**

A cross-sectional design based on mixed-method approach was conducted in two schistosomiasis endemic districts in Ghana, Lower Manya-Krobo and Shai Osudoku districts.

**Methods:**

The study surveyed 856 women of reproductive age and conducted focused group discussions with 88 opinion leaders, adolescent girls, and women of reproductive age in 20 communities along the Volta Lake. A descriptive analysis, Chi-square test, and inferential statistics were employed on the survey data using STATA SE-18, while thematic analysis was used for the qualitative content using NVivo 20.

**Results:**

The findings revealed that many of the participants had poor knowledge, attitudes, and practices related to FGS, with the Lower Manya-Krobo exhibiting slightly worse scores than Shai Osudoku. Only 17.9 % had heard of FGS before among the 856 participants, and factors such as age above 40 years (AOR6.91, 95 %CI:1.98, 11.84, p < 0.00), 6–10 years stay in community (AOR3.22, 95 %CI:0.49, 5.94, p < 0.00), farmers (AOR9.69, 95 %CI:6.23, 13.17, p < 0.00) statistically predicted knowledge in SOD. Compared to LMK, all age groups, farmers (AOR15.95, 95 %CI:11.72, 20.24, p < 0.00), and heard of FGS (AOR-5.42, 95 %CI: 8.51, −2.34, p < 0.00) influenced their knowledge, attitudes, and practices. The poor knowledge, attitudes, and practices and financial constraints were major barriers that led to self-treatment and delay in seeking care from hospitals.

**Conclusion:**

The study highlighted significant gaps in KAP towards FGS among women in LMK and SOD districts. These findings reflect broader challenges observed in other schistosomiasis-endemic regions, where inadequate education, financial barriers, and limited healthcare infrastructure hinder the effective management of FGS. Addressing these gaps is crucial to improving reproductive health issues and calls for enhanced community-based health education, improved healthcare facility capabilities and resources, training healthcare professionals, and the development of context-specific strategies to address the identified gaps to improve FGS case reporting and management.

## 1 Introduction

Female Genital Schistosomiasis (FGS) is a neglected tropical disease that poses a silent yet significant threat to women and adolescent girls in Schistosomiasis-endemic areas [1,2], affecting over 56 million individuals in sub-Saharan Africa (SSA) with a prevalence ranging from 10 % to 75 % [1,[3], [4], [5]]. The national FGS prevalence for Ghana is unknown, however, Yirenya-Tawiah et al., reported 10.6 % along the Volta basin in 2011 and Kaur et al., reported 79.5 % along the Weija Lake in the Greater Accra Region, Ghana in 2013 [5,6]. Women's daily activities, such as fetching water and farming near contaminated freshwater expose them to FGS, often without their knowledge [7,8]. FGS arise from untreated urogenital Schistosomiasis caused by *Schistosoma haematobium,* affecting the reproductive system [2,9] and leading to distinctive genital lesions [10,11]. Antigens released by the adult worms found in the pelvic veins and viable *S*. *haematobium* eggs lodge in the genital tract, triggering a chronic inflammatory response leading to scarring and fibrosis of the genital tissues, hence, lesion formation [[12], [13], [14]]. Symptoms like vaginal itch, vaginal discharge, and pelvic pain are frequently misdiagnosed as sexually transmitted infections [[15], [16], [17], [18]], contributing to delays in treatment and exacerbation of complications.

FGS is linked to serious health consequences, including infertility, miscarriage, ectopic pregnancy, and an increased risk of Human Immunodeficiency Virus (HIV) and Human papillomavirus (HPV) [[19], [20], [21], [22], [23]]. Additionally, the disease has psycho-social impact such as depression and stigma, further diminishing the quality of affected women [24]. Despite its significant health burden [16,19,21,23,25], FGS remains largely ignored by policymakers and public health experts [[26], [27], [28]]. Community members, particularly women of childbearing age, are often unaware of the disease [[29], [30], [31]], and primary healthcare facilities in endemic areas lack the capacity to diagnose and manage it effectively [10,11]. This lack of awareness, coupled with disinformation and cultural beliefs, hinders early detection and treatment [29,30].

The neglect of FGS underscores a critical gap in public health knowledge and intervention. While schistosomiasis is relatively well-known, FGS has not received comparable attention, leaving affected women vulnerable to severe health outcomes [14]. As we move through the sociocultural landscape, it becomes clear that FGS is intertwined with personal beliefs, educational level, and social systems [12,29,30]. Addressing this knowledge gap is vital for improving early diagnosis and comprehensive care, as well as for developing effective public health strategies to combat the disease.

This study aims to fill the existing knowledge gaps by exploring the knowledge, attitudes, and practices surrounding FGS among women of reproductive age in two schistosomiasis-endemic districts in Ghana. By understanding these factors, the research seeks to inform targeted interventions that improve women's health outcomes and foster community well-being in endemic areas [32]. With only few previous studies on community awareness of FGS in SSA, this research this research is critical step toward addressing the pervasive and overlooked burden of FGS [29,30].

## 2 Methods

### 2.1 Study design

A cross-sectional study based on a sequential mixed-method approach was employed [[33], [34], [35]]. The sequential mixed method allows the integration and synthesis of the quantitative and qualitative data from the participants to unearth the factors that influence participants’ FGS knowledge, attitude, and perception (KAP) [35,36]. The qualitative study employed the phenomenological approach to allow respondents to share their lived experiences and social context through Key Informant Interviews (KII) and Focused group discussion (FGD) [35,[37], [38], [39], [40]].

### 2.2 Study setting

The Lower Manya-Krobo Municipal (LMK) occupies a land area of 1476 square km in the Eastern Region [41], and the Shai Osudoku District (SOD) covers a land size of 968.361 square km in the Greater Accra Region of Ghana [42]. The districts were purposively selected based on historical data that shows their endemicity in schistosomiasis and have communities along the Volta Lake [29,41,42]. Most households in both districts depend on Volta Lake for economic, recreational, and domestic purposes. Artificial dams and ponds of varying sizes are used for irrigation and watering livestock such as the Natriku dam [42]. Rice farming, trading, and fishing are some of the main occupations [41].

### 2.3 Study population and sample size

The study targeted women aged 16–49 years for the survey, with qualitative data gathered from community leaders, adolescent girls, and women groups. The survey sample size was estimated at 856 women (428 per district) using Pearson's chi-square test, assuming 50 % baseline FGS knowledge and a 10 % prevalence difference. Alongside, 20 leaders, 36 girls and 32 women were sampled for the qualitative study.

### 2.4 Sampling for the survey, KII and FGS

A multi-stage probability sampling approach was used to select ten enumeration areas, and ten communities based on the 2021 Ghana Population and Housing Census as the primary sampling units in both districts. We then estimated the proportion of women of reproductive age in each community and proportionally allocated samples to account for the different population size and representativeness. A simple random sampling was then employed to select from each community for the survey whiles a purposively sampling was employed for the KII and FGD.

### 2.5 Survey tool, data collection and quality control

A structured questionnaire and an interview guide based on literature and a conceptual framework was designed and sectioned into four areas: Section A included demographic characteristics (age, educational level, ethnicity, occupation, years of residency in community, heard of FGS, and health-seeking behaviors) [[29], [30], [31],[43], [44], [45], [46]]. Section B had seven questions on knowledge (cause, transmission, symptoms, diagnosis, complications, treatment, and prevention). Attitudes and Practices towards FGS had 7 and 6 questions on a 5-point Likert Scale in sections C and D (Supplementary file 1a_Survey questionnaire). The survey data was administered by trained data collectors and collected via the Kobo Collect humanitarian Toolbox and voice recording and field notes were used for the qualitative data [[29], [30], [31]].

A semi-structured interview guide was used for the KII and FGD (Supplementary File 1b_Interview guide). A blend of Ga-Dangme, Twi, and English was used for the discussion to allow participants to express themselves in a language they were comfortable with.

The questionnaire and interview guide were pre-tested on ten randomly selected women in a community that was not included in the study. This helped fix issues with unclear wording, interpretation, improved internal validity and reliability and duration of the survey and FGD before the final administration. Data collectors made a house-to-house visit for the face-to-face interview with all eligible participants.

### 2.6 Data management

#### 2.6.1 Data cleaning

The captured data was downloaded in Microsoft Excel format and exported into STATA SE-17 for cleaning (e.g., removing missing data and errors). Six observations with missing values were dropped. We renamed and labelled all variables and reversed scales where needed.

#### 2.6.2 Variables measurement

We considered respondents' age, educational level, ethnicity, occupation, religion, years of stay in the community, first place of call when ill and heard of FGS before as independent variables. The dependent variables were FGS knowledge, attitudes towards FGS and practices towards FGS. These dependent variables are composite scores generated from summing up participants’ responses.

#### 2.6.3 FGS knowledge among respondents

FGS knowledge was assessed as a percentage score based on seven key questions, including awareness of FGS, its causes, transmission modes, symptoms, treatment, prevention and complications. Each correct response scored one point, and the maximum expected score was 15. The total score was converted to a percentage by diving it by the number of scores and multiplying by 100. The mean scores were 44.6 % for Lower Manya-Krobo and 46.4 % for Shai Osudoku, with scores below the mean classified as “poor knowledge” and above as “good knowledge” [47].

#### 2.6.4 Attitude and practices toward FGS

Attitude toward FGS were measured as a percentage score on seven Likert scale questions (ranging from 1- strongly disagree to 5-strongly agree), covering perceptions about FGS seriousness, causes, treatment, and stigma. The total score was divided by the maximum possible score (35), multiplied by 100, and categorized into “poor” or “good” based on the mean percentage score for each district. Practices towards FGS were similarly assessed using a “Yes” and “No” questions (0-No and 1-Yes), with a total score converted into a percentage and mean scores of 29.7 % and 29.4 % computed for Lower Manya-Krobo and Shai Osudoku districts, respectively.

### 2.7 Data analysis

#### 2.7.1 Quantitative

Descriptive statistics were used to analyze participants’ background characteristics and FGS KAP, while Pearson Chi-square assessed the significance between dependent and independent variables. Bivariate and multivariate linear regression models identified variables influencing FGS KAP, with multivariable models focusing on biologically plausible and statistically significant (p < 0.05) variables.

#### 2.7.2 Qualitative

Verbatim transcription of KII and FGD audio recordings were verified by the first author for accuracy, and a codebook was developed was developed using the interview guide to generate deductive codes. Transcripts were coded using both deductive and inductive approaches to capture new codes and themes without losing their original meanings [35,48]. The finalized codebook guided thematic analysis, with transcripts imported into NVivo 12 (Lumivero, MA, USA) [49], where codes were merged into parent themes based on identified relationships.

## 3 Results

### 3.1 Participants background characteristics

A total of 856 (428 per district) were included in this analysis with a mean age of 29.4 with a standard deviation of ±7.2. In LMK Municipal, 52.6 % had primary, 64.9 % were Ewes, 54.7 % were traders, 59.1 % of participants considered the hospital/clinics as the first place of call when ill and only 17.1 % of participants have heard of FGS before. In SOD, 55.6 % had completed primary education, 77.3 % hail from the Ga-Dangme ethnic group, 35.8 % were traders, 59.4 % first point of call when ill was the hospital/clinics and approximately 18.7 % of participants had heard of FGS before (Table 1).

**Table 1:** Description of participants’ background characteristics by district of study.

Variables	Lower Manya-Krobo Municipal (n = 428)		Shai Osudoku District (n = 428)		Total respondents (n = 856)		P-value
	Freq	%	Freq	%	Freq	%	
**Mean age (Sd)**	29.7 (±6.98)		29.3 (±7.42)		29.5 (±7.20)		
**Age group**							

<20 years	38	8.8	53	12.4	91	10.6	
21–30 years	202	47.2	198	46.3	400	46.7	0.41
31–40 years	151	35.3	141	32.9	292	34.1	
41–50 years	37	8.6	36	8.4	73	8.5	

**Educational level**							

No formal education	75	17.5	56	13.1	131	15.3	
Primary	225	52.6	238	55.6	463	54.1	0.61
Secondary	121	28.3	131	30.6	252	29.4	
Tertiary	7	1.6	3	0.7	10	1.8	

**Ethnicity**							

Akan	7	1.6	5	1.2	12	1.4	
Ewe	278	64.9	89	20.8	367	42.9	0.00∗
Ga-Dangme	133	31.1	331	77.3	464	54.2	
Others	10	2.3	3	0.7	13	1.5	

**Occupation**							

Artisan	29	6.8	53	12.4	82	9.6	
Trader	234	54.7	153	35.8	387	45.2	
Farmer	68	15.9	70	16.4	138	16.1	0.00∗
Government worker	9	2.1	3	0.7	12	1.4	
Unemployed	80	18.7	108	25.2	188	21.9	
Others	8	1.9	41	9.6	49	5.7	

**Religion**							

Christianity	411	96.0	423	98.3	834	97.4	0.01∗
Other religion	17	4.0	5	1.7	22	2.6	
First place of call when ill							
Self-medication	11	2.6	32	7.5	43	5.0	0.00∗
Clinic/HC/Hospital	253	59.1	254	59.4	507	59.2	
Chemical shop/Pharmacy	164	38.3	142	33.1	306	35.8	

**Heard of FGS**							

Yes	73	17.1	80	18.7	153	17.9	0.53
No	355	82.9	348	81.3	703	82.1	

**Years of stay in the community**							

0–5 years	121	28.3	122	28.5	243	28.4	
6–10 years	132	30.8	127	29.7	259	30.2	0.93
Above 10 years	175	40.9	179	41.8	354	41.4	354

Key: P < 0.05 = ∗; M = mean; Sd = standard deviation, (%) = percentage.

### 3.2 Participants’ knowledge, attitudes, and practices

Supplementary File 2_Table 1 present the responses relative to the knowledge score which covered the causes, symptoms, mode of transmission, treatment, and prevention. All the respondents (99.8 % in LMK and 98.4 % in SOD) knew a parasitic worm in the water bodies caused the disease. Contact with water bodies and blood in urine were the main modes of transmission and signs and symptoms respondents gave. Overall, 49.3 % indicated praziquantel as the medicine for the treatment FGS.

The mean score on knowledge, attitudes and practices were significantly different between the districts (p < 0.00) as presented on Table 2. Most participants in LMK scored lower below the mean score, hence, 57.2 %, 77.3 % and 58.4 % for poor knowledge, attitude and practices respectively as compared to participants in SOD. The participants from the two districts were significantly different (p < 0.00).

**Table 2:** Description and categorization of mean scores for knowledge, attitude, and practices towards FGS among women of reproductive age.

Variable	Lower Manya-Krobo n = 428 (%)		Shai Osudoku n = 428 (%)		Total respondents n = 856 (%)		P-value
	Frequency	Percentage	Frequency	Percentage	Frequency	Percentage	
**Knowledge M(Sd)**	44.6 (±13.9)		46.4 (±11.8)		45.5 (±12.9)		
Poor	245	57.2	205	47.9	450	52.6	0.00∗
Good	183	42.8	223	52.1	406	47.4	

**Attitude M(Sd)**	48.8 (±3.8)		50.2 (±5.8)		49.5 (±4.9)		

Poor	331	77.3	262	61.2	593	69.3	0.00∗
Good	97	22.7	166	38.8	263	30.7	

**Practices M(Sd)**	29.8 (±17.9)		29.4 (±17.4)		29.5 (±17.7)		

Poor	250	58.4	279	65.2	529	61.8	0.04
Good	178	41.6	149	34.8	327	38.2	

*Key: The chi-square test of association was statistically significant at p* < *0.05,* M = mean; Sd = standard deviation, (%) = percentage.

The focused group discussion (FGD) among the opinion leaders, women group and adolescent girls revealed the misinformation, perception and opinions about FGS which depict the depth of the poor knowledge, attitudes, and practices as quoted below.

"It is when you urinate blood because of contamination of our river bodies. That is when toxic materials in the river contaminate it. For instance, the river here sometimes overflows, and children go in to play; as a result, toxic substances can enter their bodies, and that causes that infection" … (LMK/FGD_OP_004).

*"When we bathe or drink river water which is contaminated, we are being infected with some microorganisms; then later you will start urinating blood"* … *(LMK/FGD_OP_003).*

"Microorganisms find their way into your body when you sit in the river naked. When you bathe at the banks of the river, sand and other microorganisms can enter your vagina, which can cause bilharzia." … (FDG/SOD_AD_005).

"If you are using one sponge with someone, who has it, through that you can get it. Because when bathing, the person will bathe her private part. Some of the bacteria will be on the sponge, and you also bathe with it, you can be affected" … (FGD/SOD_WG_004).

### 3.3 Factors influencing FGS knowledge, attitudes, and practices

In LMK municipal, a percentage increase in all ages and farmers positively predicted the **FGS knowledge** score. Older participants had better FGS knowledge, and scores compared to younger ones such as above 40 years (AOR:7.50, 95 %CI:1.78, 13.23, p = 0.01) were observed. Again, farmers exhibited better understanding of FGS (AOR:15.98, 95 %CI:11.72, 20.24, p < 0.00) as compared to other occupations. Similarly in SOD, older participants above 40 years, (AOR:6.91, 95 %CI:1.98, 11.84, p < 0.00) showed better knowledge of FGS. Also, farmers (AOR:9.69, 95 %CI:6.23, 13.17, p < 0.00) and those who have resided for 6–10 years in the community (AOR:3.22, 95 %CI:0.49, 5.94, p < 0.00) had better understanding of FGS (Supplementary File 3_Table 1).

Participants who had completed secondary education had better **attitude towards FGS** (AOR:2.69, 95 %CI:0.71, 4.66, p < 0.00) but participants who work in the formal sector exhibited poor attitudes (AOR: 9.87, 95 %CI: 15.41, −4.32, p = 0.01) as compared to other occupations in LMK. In the SOD district, older participants had an increased attitude towards FGS compared to those younger. Again, participants, with primary education, traders and those who have never heard of FGS had a decreased score on **attitude towards FGS** (Supplementary File 3_Table 2).

In LMK, participants who had resided more than ten years in the community (AOR:3.90, 95 %CI:2.17, 5.63) and those who completed secondary education (AOR: 3.03, 95 %CI: 5.28, −0.78, p < 0.00) were associated with good **Practices towards FGS**. However, in SOD, higher educational status had better **Practices towards FGS**. We also observed, resident above ten years in the community (AOR:2.95, 95 %CI:1.18, 4.73, p < 0.00) had better **Practices towards FGS** (Supplementary File 3_Table 3).

The FGD also revealed some factors influencing FGS KAP in the study areas as shown in the quotes below. These factors ranged from lack of community awareness of schistosomiasis MDA, adverse reaction of praziquantel, no MDA implementation and lack of community education about the disease.

“In the past, whenever it was time for the distribution of the schistosomiasis medicine in our communities by VRA and Ghana health service, the nurses and some community volunteers will give education about the disease. This has stopped happening in a long time” … (FGD/SOD_OP_009)

“In our community, we all bathe, wash, and fetch water from the river to bring home every day since we were young. We know that we urinate blood when we go into the river, but that is the water we have” … (FGD/SOD_WG_008)

“I have been a community volunteer before to give those medicine to my community people. That time, they give us poles to measure height of people, T-shirts, and posters of the disease. Now we do not even hear anything about the distribution anymore” … (FGD/LMK_OP_005).

“The last time they gave us the medicine in school, some of the students got sick after taking it. Me, I did not take the medicine because I was afraid, I will also get sick” …. (FGD/LMK_AD_011)

### 3.4 Barriers to seeking care and perception of health facility's capacity to manage FGS

From survey and the FGDs, financial constraints and beliefs emerged as barriers to seeking care respectively. Table 3 shows that 420 (98.1 %) respondents in each district indicated financial constraints as the major barrier to seeking care. This led many to indulge in self-medication, resulting in the purchases of drugs from community drug stores instead of going to health facilities in both districts. About 401 (94.4 %) and 394 (92.3 %) of respondents in LMK and SOD indicated receied needed services when they visit the primary healthcare (PHC) level such as the Community-based Health Planning and Services (CHPS) compound respectively. However, the FGD revealed the absence of some healthcare services i.e. laboratory services, at the CHPS were the major barriers participants from both districts indicated. Most participants who seek care from the PHC were referred to other facilities because there was no laboratory and tools to diagnose FGS and praziquantel for treatment. This contributed to the reliance on homemade remedies for treating gynecological symptoms that could be due to FGS. Participants in both districts suggested measures to improve awareness and knowledge about FGS and reduce the impact of the disease among adolescent girls and women of reproductive age in endemic areas. The most common suggestion was for the Volta River Authority (VRA) to desilt the canal, remove weeds and snail control. They also suggested that VRA continues with the schistosomiasis screening and MDA they organize sometimes. Lastly, they suggested healthcare workers educate the community about FGS.

"*People also go to traditional healer to make medicine for them so that the blood will not come again. They go to our old men to prepare home medicine for the girls*". *(LMK/FGD_AD_003)*

“It is expensive to go to the hospital now especially if you do not have health insurance. Then, you do not get all the services you want, only to be transferred to another hospital. The transport fare also increases the expenses.”(FGD/SOD_OP_014)

“Here, even if you want to go to the clinic, the roads are very bad, so it is the motorcycles (okada) that can go on that road, and they charge very expensive. If you don’t have health insurance, then it becomes expensive to go to the clinic”. (LMK/FGD_WG_010)

"People buy the medicine at that drug store to protect themselves so that the blood will stop after swimming in the lake. I do not know if the same medicine can be used for FGS" … (LMK/FGD_AD_002).

"*They cut the back of the neem tree, put it in a saucepan, pour water, put it on fire, allow it to boil, then later drain the liquid into a bottle and be drinking it. That will stop the vaginal discharge" … (FGD/SOD_AD_006).*

**Table 3:** Barriers to health seeking behaviours among respondents in Lower Manya-Krobo and Shai Osudoku Districts.

Variable	Lower Manya-Krobo Municipal (n = 428)		Shai Osudoku District (n = 428)		Total respondents (n = 856)		P_value
	Frequency	Percentage	Frequency	Percentage	Frequency	Percentage	
**Time travel Facility**							
Below 30 min	409	95.6	403	94.14	812	94.9	0.62
31–60 min	19	4.4	23	5.4	42	4.9	
Above 60 min	0	0	1	0.23	1	0.12	
Don't know	0	0	1	0.23	1	0.12	

Means of Transport							
Walking	61	14.3	89	20.8	150	17.5	0.00
Bicycle	2	0.5	0	0	2	0.23	
Motorcycle	230	53.7	307	71.7	537	62.7	
Taxi	17	3.9	23	5.4	40	4.7	
Trotro (commercial transport)	117	27.3	8	1.9	125	14.6	
Own car	1	0.23	1	0.23	2	0.23	

**Barriers to seeking care**							
Cultural belief	4	0.9	2	0.5	6	0.7	0.28
Financial issues	420	98.1	420	98.1	840	98.1	
Distance to facility	4	0.9	3	0.7	7	0.8	
others	0	0	3	0.7	3	0.4	

**Receive needed care at facility**							
Yes	401	94.4	394	92.3	795	93.3	0.22
No	24	5.6	33	7.7	57	6.7	

## 4 Discussion

The study revealed inadequate knowledge, poor attitudes, and misguided practices regarding FGS, driven by misinformation and a lack of awareness among participants. While there was a general awareness about urogenital schistosomiasis, understanding of its progression to FGS was limited. Participants in Shai Osudoku demonstrated greater FGS knowledge compared to those in Lower Manya-Krobo. This research highlighted the depth of FGS knowledge, attitudes, practices, and their influencing factors in the study area.

### 4.1 Knowledge of female genital schistosomiasis

The study revealed a generally poor knowledge about FGS, with 57.2 % of participants in LMK and 47.9 % in SOD scoring below average on knowledge. Although a greater proportion of participants were aware that FGS is caused by a parasitic worm, about 50 % were not aware of the drug for treatment (i.e. praziquantel). Similar findings have been confirmed by other studies [50,51]. Additionally, other recent studies in Tanzania and Ghana revealed a greater percentage of participants had never heard of FGS and perceived FGS as some reproductive conditions [29,30,51,52]. Participants reported of no health education nor educational materials on FGS in communities nor health facilities. The participants again indicated the screening and awareness campaign which were usually implemented by the VRA and Ghana Health Service has stop. For more than three decades Ghana Health Service and NTD program uses schistosomiasis MDA and health education as key control and elimination strategies [[53], [54], [55], [56]]. However, according to the 2021 to 2025 Ghana NTD master plan, there is no specific budget line for NTDs resulting in the program's lack of printed Information, Education, and Communication (IE&C) materials on NTDs [57]. As a result of funding constraints and a lack of resources, the Ghana Health Service and the NTD program have fallen short of their goal of eliminating the disease among endemic areas. This presents a hurdle to achieving the ending NTDs by 2030 [58]. Interestingly, comparing older participants above 40 years and farmers to younger ones, the older ones demonstrated significant knowledge, which could be due to the long existence of the disease in endemic area.

### 4.2 Attitudes towards female genital schistosomiasis

Significant gaps in attitudes towards FGS was also shown with 77.3 % and 61.2 % of participants demonstrated poor attitudes in LMK and SOD respectively. Factors influencing attitudes include age, education, and occupation with older participants generally displaying more positive attitudes. However, participants in formal sectors exhibited poorer attitudes, likely due to less direct engagement with FGS-related health issues compared to those in the farming communities. A study in Kenya reported similar findings where rural populations often have stronger attitudes toward addressing schistosomiasis due to higher exposure to water sources that serve as transmission sources. The poor attitudes reflect the limited prioritization of FGS in healthcare settings, where it is often viewed as a lesser concern compared to other gynecological conditions. A recent study in Nigeria observed how HCWs expressed reluctance to diagnose and treat FGS due to lack of training and resources [4,59].

### 4.3 Practices towards female genital schistosomiasis

The study found that 58.4 % of participants in LMK and 65.2 % in SOD reported poor practices, frequently opting for homemade remedies over formal healthcare. Critical barriers included limited access to diagnostic tools, essential drugs like praziquantel, and financial constraints, despite the availability of national health insurance at some primary healthcare (PHC) levels. A recent study highlighted alarming gaps in the prevention and treatment of FGS in low-resource settings. These results align with findings from Malawi, Tanzania and Ghana, where similar logistical and financial hurdles impede access to formal FGS care [29,30,52,60]. Notably, while participants recognize water sources such as Volta Lake as the primary source of infection, they faced practical difficulties avoiding its contact due to domestic and commercial reliance on these waters. Many resorted to traditional healers or self-medication for symptoms like blood in urine and vaginal discharge, underlining the urgent need for improved PHC delivery.

### 4.4 Factors influencing knowledge, attitudes, and practices

A recent study explored how participants’ background characteristics, such as age, education, occupation, and length of community residence, impacted their knowledge, attitudes, and practices regarding FGS. Key findings revealed that older individuals and those with higher education levels had significantly better KAP scores. This trend aligns with prior studies across SSA, showing that these groups are more engaged in healthcare and preventive measures. Notably, participants living in their communities for over ten years exhibited improved practices, likely due to increased exposure to Mass Drug Administration (MDA) campaigns. Conversely, individual with lower education particularly in LMK, demonstrated poorer attitudes and practices, highlighting the critical need for targeted education programs [50,61].

This study emphasized the importance of bridging these gaps to prevent an increase in FGS cases and complications. A promising intervention, the FGS Accelerated Scale Together (FAST) project, has been implemented in Ghana and Madagascar [62]. The project aims to reduce FGS-related morbidity by integrating diagnosis and treatment into routine health services, enhancing community awareness, training healthcare workers, and conducting annual MDA campaigns. These efforts reflect a vital step toward combating FGS through comprehensive, systemwide approaches [62].

### 4.5 Barriers to seeking health care

The data from both districts consistently revealed financial constraints as the primary barrier to seeking healthcare with 98.1 % of respondents in both districts citing cost of care and travel as a major issue. Majority of the respondents relied on motorcycles for transport, especially in LMK 53.7 % and 71.7 % in SOD, with poor roads access and high transport cost exacerbating healthcare access challenges. Notable service gaps particularly the lack of diagnostic tools and laboratory services at the CHPS compound, influenced frequent referrals and self-medication, even though majority, 93 % reported receiving essential care at the PHC facilities. Participant also reported of their reliance on cultural beliefs and traditional remedies such as the neem bark infusions and traditional healers as coping mechanism.

### 4.6 Strengths and limitations of the study

The study lays the foundation for developing interventions to address the identified gaps holistically. It is among the few studies to use a mixed method approach in sub-Saharan Africa at the time of this publication.

We did not include parasitological and clinical surveillance for FGS in this study, as such, FGS prevalence in the study area is unknown. The framing of the survey and qualitative questions and how they were asked may have influenced participants’ responses.

## 5 Conclusions and recommendations

This study highlights significant gaps in knowledge, attitudes, and practices regarding FGS among women in two endemic districts along Ghana's Volta Lake. These findings mirror challenges in other schistosomiasis-affected regions, where inadequate education, financial constraints, and weak healthcare infrastructure hinder effective FGS management.

Addressing these gaps requires robust public health strategies rooted in behavior change communication to promote early diagnosis and treatment. The study recommends that the Ghana Health Service and National NTD program implement context-specific education initiatives, leveraging local media to raise awareness and empower communities about FGS. Additionally, media campaigns, posters, and flyers should publicize FGS, while community engagement during MDA campaigns is crucial to boost participation and acceptance. Strengthening these efforts could significantly reduce the burden of FGS in Ghana.

## What this study adds

• Lay a foundation for the development of context-specific intervention to address identified gaps
• Confirms the interplay between socio-cultural beliefs, knowledge, attitudes, and practices towards FGS which should be considered for holistic solutions.

## Implication for policy

• Community-based health education campaigns based on locally available media to increase FGS awareness among at-risk populations should be implemented like health campaigns for malaria, HIV and COVID-19 by policy makers.
• Resourcing the primary healthcare facilities and healthcare workers within rural areas to diagnose and manage FGS cases.

## Ethical approval

The Biomedical Research Ethics Committee (BREC) of the University of KwaZulu-Natal (Certificate number BREC/00005309/2023) and the Ethics Review Committee of the Ghana Health Service (Certificate number GHS-ERC:008/02/23) issued clearance for this study. Stakeholder permission was obtained from the National Neglected Tropical Diseases Program (NTDP) of Ghana Health Service (GHS), Regional, Districts and community authorities of the Lower Manya-Krobo Municipal and the Shai Osudoku Districts. All ethical principles were adhered to. Participants’ consent for publication is not applicable.

## Authors contribution

CDT, AKM, JRN, and TGG conceptualized the study. Methodology, data collection, data curation and analysis, original draft, writing (review and editing) was done by CDT. Supervision, review and editing was done by AKM, JRN, and TGG. The final draft was done by CDT and approval was accepted by all authors.

## Data availability statement

The qualitative data cannot be shared publicly because it contains identifiers of participants. The survey data will be made available on request to the corresponding author.

## Funding

The authors declared no specific funding or grant for this study. However, this research was made possible through a HEARD PhD scholarship at the University of KwaZulu-Natal (UKZN), funded by the Swedish International Development Agency (SIDA). Any opinion, findings, and conclusions or recommendations expressed in this material are those of the authors and do not necessarily reflect the view of HEARD, UKZN, and SIDA.

## Competing interest

The authors declared no conflict of interest that could influence the study results reported in this manuscript.
