# Supplementary material for: Female genital schistosomiasis in Ghana: An exploration of knowledge, attitudes, and practice among women of reproductive age
Source: Public Health Pract (Oxf). 2025 Jun 26;10:100632. doi: 10.1016/j.puhip.2025.100632 (PMC12274308; doi:10.1016/j.puhip.2025.100632)
Supplement: Multimedia component 1 [file mmc1.pdf]

Community FGS KAP Questionnaire

PARTICIPANTS' STATEMENT I acknowledge that I have read or have had the purpose and contents of Participants' Information Sheet read and all questions satisfactorily explained to me in a language (Twi, Damgbe, Krobo, English) I understand. I fully understand the contents and potential implications as well as my right to change my mind (i.e., withdraw from the research) even after I have signed this form. I voluntarily agree to be part of this research.

Participant consent

- Yes, I accept
- No, I decline

Enter a date and time

yyyy-mm-dd hh:mm

Baseline Survey Questionnaire (women of reproductive age). Section A: Participants background information

A1. Which is your district of residence?

- Shai Osudoku district
- Lower Manya-Krobo municipal

A2. What is the name of the sub-district you live in?

- Dodowa sub-district
- Ayikuma sub-district
- Agomeda sub-district
- Duffour sub-district
- Osudoku sub-district
- Odumase sub-district
- Akuse sub-district
- Agomenya sub-district
- Kpong sub-district
- Asitey sub-district
- Oborpa sub-district

A3. What is the name of the community you stay in?

A4. How long have you lived or stayed in this community?

____

A5. How many people are in your household? (Household: Number of people who eat from the same pot)

____

A6. What gender is the household head?

- ◯ Male
- ◯ Female
- ◯ Undisclosed

A7. What is your age in completed years?

____

A8. How many females are between the ages of 15 to 49 years in your household?

____

A9. Which is your ethnicity?

- ◯ Ga-Damgbe
- ◯ Krobo
- ◯ Akan
- ◯ Ewe
- ◯ Dagomba
- ◯ Others

A9b. If other to the above question, provide your response.

____

A10. What is the source of water for your household?

- ☐ Pipe (tap) water
- ☐ Borehole
- ☐ Dam water
- ☐ Rain water
- ☐ River / Stream / Lake
- ☐ Dug well
- ☐ Other

A11. Which religious group do you belong to?

- ◯ Christianity
- ◯ Moslem
- ◯ African Traditional Religion
- ◯ Buddha / Krishna / Eckankar
- ◯ Pegan
- ◯ Others

A11b. If Others to the above question, provide your response.

____

A12. What is your highest level of education?

- ◯ No formal education
- ◯ Primary school
- ◯ Secondary school
- ◯ Tertiary

A14. What is your occupation (work you do for living)?

- ◯ Unemployed
- ◯ Trading
- ◯ Farmer
- ◯ Civil /public servant
- ◯ Artisan
- ◯ Others

A14b. If others to the above question, provide your response

____

A15. How far is your house from the Lake / river / stream / dam in your community?

- ◯ Less than 1 kilometer
- ◯ Greater than 1 kilometer
- ◯ Don't know

A16. Have any of your household members ever swim or bath in the Lake / river / stream / dam in your community?

- ◯ Yes
- ◯ No
- ◯ Don't know

Participants health seeking behaviour

A17. Where is the first place of call when any of the household member is not well?

- ◯ Self-medication
- ◯ Chemical / drug shop
- ◯ Pharmacy shop
- ◯ Private hospital
- ◯ CHPS / Clinic, Health center / Polyclinic / District hospital
- ◯ Traditional herbal clinic
- ◯ Spirital healer
- ◯ Alternative medicine (homeopathy)
- ◯ Others

A17b. If others to the above question, provide your response.

A18. How long does it take you to get to the health facility if you choose to go there for care?

- ◯ Less than 15 minutes
- ◯ 15 - 30 minutes
- ◯ 31 - 60 minutes
- ◯ More than 1 hour
- ◯ Don't know

A19. What means of transport is usually used by most household members to get to the health facility the household normally uses?

- ◯ Walking
- ◯ Bicycle
- ◯ Motor cycle or bike
- ◯ Taxi
- ◯ Trotro
- ◯ Own car
- ◯ Canon
- ◯ Others

A19b. If others to the above question, provide your response.

A20. Is there any disease that you believe can only be treated by traditional healers?

- ◯ Yes
- ◯ No
- ◯ Don't know

A20 b. If 'Yes" to the above question, name the disease? Skip this question if participant answers "No" to the above question.

A21. Is there any disease that you believe can only be treated only by spiritual healers?

- ◯ Yes
- ◯ No
- ◯ Don't know

A21 b: If 'Yes" to the above question, name the disease. Skip this question if participant answers "No" to the above question.

A22. Is there any disease that you believe can only be treated in the health facility?

- ◯ Yes
- ◯ No
- ◯ Don't know

A22 b: If 'Yes" to the above question, name the disease. Skip this question if participant answers "No" to the above question.

A23. Is there any disease that you believe can only be treated by alternative medicine (homeopathy)?

- ◯ Yes
- ◯ No
- ◯ Don't know

A23 b. If 'Yes" to the above question, name the disease. Skip this question if participant answers "No" to the above question.

A24. What are the main barriers that prevent community members from seeking medical care services from health facility?

- ◯ Cultural beliefs
- ◯ Financial issues
- ◯ Geographical (distance to the health facility)
- ◯ Others

A24b. If others to the above question, provide your response.

____

A25. Do you receive the needed healthcare or services when any member of your household visits the health facility in your community?

- ◯ Yes
- ◯ No
- ◯ Don't know

A26. Have you subscribed to the National Health Insurance Scheme?

- ◯ Yes
- ◯ No
- ◯ Don't know

A26 b. If 'No' to the question above, how much out of pocket money do you usually spend when you visit the health facility for healthcare?

- ◯ Less than 50 cedis
- ◯ 50 - 250 cedis
- ◯ 251 - 450 cedis
- ◯ 451 - 650 cedis
- ◯ Greater than 651 cedis

A27. Has any of your household members ever urinated blood before?

- ◯ Yes
- ◯ No
- ◯ Don't know

A27 b. If 'Yes' to the question above, how was the person treated?

- ☐ Treated in health facility
- ☐ Treated using herbal medicine
- ☐ Treated by spiritual healer
- ☐ Self-medication
- ☐ Treated using alternative medicine
- ☐ Other

A27 bi. If other to the above question, provide your response.

____

Section B contains question on knowledge, attitude and practices. Please choose a response that best answers the 7 question on knowledge

---

B1. Have you ever heard about urinating blood disease before?

- ◯ Yes
- ◯ No
- ◯ Don't know

B1 b. If 'Yes" to the above question, what is the local name for urinating blood?

____

B1c. Have you ever heard about female genital schistosomiasis (FGS) or urinating blood disease in females before?

- ◯ Yes
- ◯ No

B2. What causes Female Genital Schistosomiasis? (Select as many as may apply)

- ☐ Worms / germs in the lake / river / stream / dam / stream etc
- ☐ Bacteria / germs from sexually transmitted diseases (STDs)
- ☐ Poor personal hygiene
- ☐ Spiritual
- ☐ Mosquito bite
- ☐ Food poison

B3. Through what means does someone get infected with female genital schistosomiasis? (Select as many as may apply)

- ☐ Swimming in dirty water infested with worms
- ☐ Drinking dirty water from the lake
- ☐ Sexual intercourse
- ☐ Eating contaminated froom
- ☐ Fetching water from the Lake
- ☐ Washing cloths in the Lake / river / dam / stream
- ☐ Other

B3b. If other to the above question, provide your response

____

B4. What are the signs and symptoms of female genital schistosomiasis? (Select as many as may apply)

- [ ] Blood in urine
- [ ] Vagina discharge
- [ ] Abdominal and pelvic pain
- [ ] Contact bleeding
- [ ] Bleeding after sexual intercourse
- [ ] Vaginal itching
- [ ] Menstrual pains
- [ ] Pale palm or eyes (anaemia)
- [ ] Other
- [ ] Prefer not to answer

B4b. If other to the above question, provide your response

____

B5. What measures can be taken to prevent female genital schistosomiasis? (Select as many as may apply)

- [ ] Avoid urinating and defecating in water bodies
- [ ] Avoid contact with infested water
- [ ] Use condom for sexual intercourse
- [ ] Avoid swimming in the Lake / river / dam / stream
- [ ] Drink a dose of praziquantel every year
- [ ] Seek early treatment in health facility
- [ ] Seek traditional herbs treatment
- [ ] Avoid multiple sexual partners (promiscuity)
- [ ] Don't know

B6. What medicine can be used to treat Female genital schistosomiasis? (Select as many as may apply)

- [ ] Hospital medicine (Praziquantel)
- [ ] Dewormer (Albendazole, Mebendazole)
- [ ] Herbal medicine
- [ ] Spiritual healing (prayer, libation)
- [ ] Healthy eating
- [ ] Good personal hygiene (WASH)
- [ ] Don't know

B7. What are the complications of female genital schistosomiasis? (Select as many as may apply)

- [ ] Infertility (childlessness)
- [ ] Stillbirth
- [ ] Abortion
- [ ] Maternal death
- [ ] Divorce
- [ ] Stigmatisation
- [ ] Shame
- [ ] Body Odour
- [ ] HIV / AIDS
- [ ] Cervical cancer
- [ ] Don't know

Section B: Questions on Attitude (Please provide an appropriate answer to the questions below by selecting your choice of response. Select the following scale; 1-Strongly disagree; 2-Disagree; 3-Neutral; 4-Agree; 5-Strongly Agree against your choice of response)

B8. Female Genital Schistosomiasis is a serious disease that affects women and young girls of reproductive age.

Strongly Disagree | Disagree | Neutral | Agree | Strongly Agree

B9. Female Genital Schistosomiasis is a disease that affects women who have constant contact with water bodies (Lake, river, dam, stream)

Strongly disagree | Disagree | Neutral | Agree | Strongly agree

B10. Female Genital Schistosomiasis is a disease that affects women who cheat on their partners

Strongly disagree | Disagree | Neutral | Agree | Strongly agree

B11. Female Genital Schistosomiasis is a spiritual disease that is caused by the gods.

Strongly disagree | Disagree | Neutral | Agree | Strongly agree

B12. Female Genital Schistosomiasis can be cured if detected early.

Strongly disagree | Disagree | Neutral | Agree | Strongly

B13. I would not tell my partner or relative if I suspect I have FGS symptoms with myself

Strongly disagree | Disagree | Neutral | Agree | Strongly agree

B14. I think there is no treatment or cure for female genital schistosomiasis

Strongly disagree | Disagree | Neutral | Agree | Strongly agree

Section B: Questions on Practices (Please provide an appropriate answer to the questions below by selecting your choice of response. Select "No" or "Yes" against your choice of response

B15. I have taken or swallowed anti-schistosome drug (praziquantel) before

Yes | No

B16. The hospital is the first place I visit when I experience a vaginal infection

No | Yes

B17. I use homemade remedies when I experience vagina infection

No | Yes

B18. I make contact with a water body (Lake, river, stream, swamp) in my routines (house chores, farming, trading).

No | Yes

B19. I have ever been diagnosed and treated for Female Genital Schistosomiasis before

No | Yes

B20. I use herbs to clean myself (doching) to prevent female Genital Schistosomiasis

No | Yes

Name of data collector

THANK YOU VERY MUCH FOR YOUR TIME AND RESPONSES
