# Supplementary material for: Female genital schistosomiasis in Ghana: An exploration of knowledge, attitudes, and practice among women of reproductive age
Source: Public Health Pract (Oxf). 2025 Jun 26;10:100632. doi: 10.1016/j.puhip.2025.100632 (PMC12274308; doi:10.1016/j.puhip.2025.100632)
Supplement: Multimedia component 2 [file mmc2.docx]

**Women FGD guide**

**The University of Kwazulu-Natal**

**School Of Nursing and Public Health, Durban, South Africa**

**Study title:** Improving knowledge and Management of Female Genital Schistosomiasis in Ghana: A Step towards Schistosomiasis Control and Elimination using an Implementation Science Approach

**Interview Guide: Community members Focused Group Discussion (FGD)**

***Introduction***: ***Thank you for consenting to participate in this interview. This study is purely for academic purposes and the interview will take between 45 minutes to 1 hour.***

**Question**

1. What do you know about female genital schistosomiasis? (Probe: cause, transmission, prevention, signs, symptoms, etc.)
2. How do you explain the healthcare-seeking behavior of our society for female genital schistosomiasis and other reproductive system infection?
3. Are there cultural beliefs attached to female genital schistosomiasis to patients in your community?
4. How would you explain the treatment, prevention, and control of female genital schistosomiasis?
5. How do you explain the social stigma of female genital schistosomiasis patients in your community?
6. How do you explain the healthcare provider’s approach to treating female genital schistosomiasis patients? (Probe for the traditional or local approach for treatment)
7. How do you explain the accessibility of health facilities in this community? (clinic, health center, and hospitals).
8. What do you think about the long-term effect female genital schistosomiasis can cause on females while there is an effective treatment?
