# Supplementary material for: Female genital schistosomiasis in Ghana: An exploration of knowledge, attitudes, and practice among women of reproductive age
Source: Public Health Pract (Oxf). 2025 Jun 26;10:100632. doi: 10.1016/j.puhip.2025.100632 (PMC12274308; doi:10.1016/j.puhip.2025.100632)
Supplement: Multimedia component 3 [file mmc3.docx]

Table 1: Participants score on variables assessing FGS knowledge

| **Description** | **Lower Manya-Krobo**  **n= 428** | | **Shai Osudoku**  **n= 428** | | **Total**  **n= 856** | |
| --- | --- | --- | --- | --- | --- | --- |
|  | **Frequency** | **%** | **Frequency** | **%** | **Frequency** | **%** |
| **Causes of FGS** |  |  |  |  |  |  |
| Parasitic worm in water | 427 | 99.8 | 421 | 98.4 | 848 | 99.1 |
| STIs | 9 | 2.1 | 9 | 2.1 | 18 | 2.1 |
| Personal hygiene | 27 | 6.3 | 55 | 12.9 | 82 | 9.6 |
| Spiritual | 0 | 0.0 | 1 | 0.2 | 1 | 0.1 |
| Mosquito bite | 0 | 0.0 | 1 | 0.2 | 1 | 0.1 |
| Food poison | 0 | 0.0 | 2 | 0.5 | 2 | 0.2 |
| **Mode of transmission** |  |  |  |  |  |  |
| Swimming in water bodies | 324 | 75.7 | 394 | 92.1 | 718 | 83.9 |
| Drinking contaminated water | 81 | 18.9 | 116 | 27.1 | 197 | 23.0 |
| Sexual intercourse | 14 | 3.3 | 16 | 3.7 | 30 | 3.5 |
| Eating contaminated food | 7 | 1.6 | 16 | 3.7 | 23 | 2.7 |
| Fetching water from water bodies | 53 | 12.4 | 55 | 12.9 | 108 | 12.6 |
| Washing inside the stream | 124 | 29.0 | 147 | 34.4 | 271 | 31.7 |
| **Signs & symptoms** |  |  |  |  |  |  |
| Blood in urine | 376 | 88.1 | 338 | 79.0 | 714 | 83.5 |
| Vagina discharge | 222 | 52.0 | 260 | 60.8 | 482 | 56.4 |
| Abdominal and pelvic pain | 33 | 7.7 | 74 | 17.3 | 107 | 12.5 |
| Contact bleeding | 23 | 5.4 | 11 | 2.6 | 34 | 4.0 |
| Vagina itch | 207 | 48.5 | 210 | 49.1 | 417 | 48.8 |
| Menstrual pain | 23 | 5.4 | 28 | 6.5 | 51 | 6.0 |
| **Treatment** |  |  |  |  |  |  |
| Praziquantel | 222 | 51.9 | 200 | 46.7 | 422 | 49.3 |
| Albendazole/Mebendazole | 1 | 0.2 | 3 | 0.7 | 4 | 0.5 |
| Herbal remedies | 15 | 3.5 | 5 | 1.2 | 20 | 2.3 |
| Prayer (spiritual) | 0 | 0.0 | 1 | 0.2 | 1 | 0.1 |
| Don’t know | 206 | 48.1 | 227 | 53.0 | 433 | 50.6 |
| **Complications** |  |  |  |  |  |  |
| Sub-fertility/Infertility | 194 | 45.3 | 284 | 66.4 | 478 | 55.8 |
| Stillbirths | 138 | 32.2 | 75 | 17.5 | 213 | 24.9 |
| Abortion | 27 | 6.3 | 15 | 3.5 | 42 | 4.9 |
| Maternal death | 11 | 2.6 | 3 | 0.7 | 14 | 1.6 |
| Divorce | 42 | 9.8 | 6 | 1.4 | 48 | 5.6 |
| Stigma | 158 | 36.9 | 119 | 27.8 | 277 | 32.4 |
| Body odour | 51 | 11.9 | 126 | 29.4 | 177 | 20.7 |
| HIV/AIDS | 1 | 0.2 | 10 | 2.3 | 11 | 1.3 |
| **Prevention & control** |  |  |  |  |  |  |
| Avoid urinating/defaecating in water | 147 | 34.4 | 243 | 56.8 | 390 | 45.6 |
| Avoid contact with water bodies | 80 | 18.7 | 87 | 20.3 | 167 | 19.5 |
| Condom use | 15 | 3.5 | 16 | 3.7 | 31 | 3.6 |
| Avoid swimming in water bodies | 407 | 95.1 | 332 | 77.6 | 739 | 86.3 |
| Annual dosage of praziquantel | 10 | 2.3 | 21 | 4.9 | 31 | 3.6 |
| Early treatment in Health facility | 121 | 28.3 | 179 | 41.8 | 300 | 35.1 |
| Herbal medicine | 6 | 1.4 | 6 | 1.4 | 12 | 1.4 |
| Don’t know | 2 | 0.5 | 1 | 0.2 | 3 | 0.4 |
