# Supplementary material for: Female genital schistosomiasis in Ghana: An exploration of knowledge, attitudes, and practice among women of reproductive age
Source: Public Health Pract (Oxf). 2025 Jun 26;10:100632. doi: 10.1016/j.puhip.2025.100632 (PMC12274308; doi:10.1016/j.puhip.2025.100632)
Supplement: Multimedia component 4 [file mmc4.docx]

Table 1: A regression analysis of background characteristics and level of knowledge of FGS in the Lower Manya-Krobo Municipal and the Shai Osudoku District

| **Variable** | **Shai Osudoku district (Case district)** | | | | **Lower Manya-Krobo municipal (Control** | | | |
| --- | --- | --- | --- | --- | --- | --- | --- | --- |
|  | **Bivariate (Crude)** | | **Multivariate (Adjusted)** | | **Bivariate (Crude)** | | **Multivariate (Adjusted)** | |
|  | **Coeff.** | **CI** | **Coeff.** | **CI** | **Coeff.** | **CI** | **Coeff.** | **CI** |
| **Age group** |  |  |  |  |  |  |  |  |
| < 20 years | Ref | Ref | Ref | Ref | Ref | Ref | Ref | Ref |
| 21-30 years | 3.01 | (-0.53, 6.54) | 1.57 | (-1.92, 5.05) | 3.68 | (-1.12, 8.49) | 5.48* | (1.13, 9.84) |
| 31-40 years | 5.19* | (1.51, 8.87) | 3.04 | (-0.88, 6.94) | 5.29* | (0.37, 10.23) | 6.48* | (1.73, 11.23) |
| 41 and above | 9.52* | (4.58, 14.45) | 6.91* | (1.98, 11.84) | 9.73* | (3.46, 16.01) | 7.50* | (1.78, 13.23) |
| **Educational** |  |  |  |  |  |  |  |  |
| None | Ref | Ref | Ref | Ref | Ref | Ref | Ref | Ref |
| Primary | -3.92* | (-7.34, -0.49) | -1.79 | (-5.00, 1.41) | -3.70* | (-7.35, -0.05) | -1.88 | (-4.93, 1.17) |
| Secondary | -5.99* | (-9.67, -2.32) | -1.50 | (-5.10, 2.09) | -2.41 | (-6.43, 1.60) | 2.08 | (-1.50, 5.66) |
| Tertiary | -6.03 | (-19.68, 7.62) | -2.00 | (-15.44, 11.44) | -7.38 | (-18.18, 3.43) | -0.51 | (-11.95, 10.94) |
| **Years of stay** |  |  |  |  |  |  |  |  |
| Below 5 years | Ref | Ref | Ref | Ref | Ref | Ref | Ref | Ref |
| 6-10 years | 4.38* | (1.46, 7.29) | 3.22* | (0.49, 5.94) | 10.89* | (7.68, 14.10) | 6.70 | (3.69, 9.70) |
| Above 10 yrs | 0.87 | (-1.83, 3.57) | -0.47 | (-3.01, 2.07) | -0.40 | (-3.42, 2.62) | -0.45 | (-3.21, 2.32) |
| **Occupation** |  |  |  |  |  |  |  |  |
| Unemployed | Ref | Ref | Ref | Ref | Ref | Ref | Ref | Ref |
| Trading | -0.57 | (-3.26, 2.12) | -0.98 | (-3.86, 1.89) | 1.43 | (-1.65, 4.52) | 0.42 | (-3.12, 3.95) |
| Farmer | 11.17* | (7.88, 14.46) | 9.69* | (6.23, 13.17) | 20.26* | (16.33, 24.19) | 15.98* | (11.72, 20.24) |
| Formal sector | 3.39 | (-9.14, 15.93) | 4.26 | (-8.86, 17.36) | 0.32 | (-8.05, 8.70) | -0.25 | (-10.29, 9.80) |
| Artisan | -2.34 | (-5.94, 1.24) | -2.67 | (-6.30, 0.96) | 2.80 | (-2.36, 7.97) | -0.14 | (-5.30, 5.02) |
| Others | -0.45* | (-8.44, -0.59) | -3.05 | (-7.05, 0.94) | 0.42 | (-8.41, 9.25) | 0.98 | (-7.59, 9.56) |
| **Heard of FGS** |  |  |  |  |  |  |  |  |
| No | Ref | Ref | Ref | Ref | Ref | Ref | Ref | Ref |
| Yes | -4.09* | (-6.95, -1.23) | -2.19 | (-4.89, 0.51) | -11.49* | (-14.84, -8.13) | -5.42* | (-8.51, -2.34) |

Table 2: A regression analysis of women background characteristics and attitudes towards FGS in the Lower Manya-Krobo Municipal and the Shai Osudoku District

| **Variable** | **Shai Osudoku district (Case district)** | | | | **Lower Manya-Krobo municipal (Control** | | | |
| --- | --- | --- | --- | --- | --- | --- | --- | --- |
|  | **Bivariate (Crude)** | | **Multivariate (Adjusted)** | | **Bivariate (Crude)** | | **Multivariate (Adjusted)** | |
|  | **Coeff.** | **CI** | **Coeff.** | **CI** | **Coeff.** | **CI** | **Coeff.** | **CI** |
| **Age group** |  |  |  |  |  |  |  |  |
| <20 years | Ref | Ref | Ref | Ref | Ref | Ref | Ref | Ref |
| 21-30 years | -4.32* | (-8.52, -0.13 | -4.32* | (-8.40, -0.25) | -2.87* | (-5.12, -0.62) | -0.87 | (-3.27, 1.53) |
| 31-40 years | -6.16* | (-10.54, -1.79) | -4.67* | (-9.25, -0.09) | -5.21* | (-7.52, -2.91) | -2.39 | (-5.01, 0.23) |
| Above 40 yrs | 2.58 | (-3.28, 8.43) | 0.03 | (-5.74, 5.79) | -1.12 | (-4.06, 1.82) | 1.21 | (-1.95, 4.37) |
| **Educational** |  |  |  |  |  |  |  |  |
| None | Ref | Ref | Ref | Ref | Ref | Ref | Ref | Ref |
| Primary | -4.72* | (-8.46, -0.97 | -4.72* | (-8.46, -0.97) | -1.70 | (0.08, -3.59) | 0.41 | (-1.27, 2.09) |
| Secondary | -0.51 | (-4.80, 3.78) | 2.55 | (-1.66, 6.76) | -3.55* | (-5.63, -1.46) | 2.68* | (0.71, 4.66) |
| Tertiary | -1.12 | (-17.05, 14.80) | 2.83 | (-12.88, 18.54) | -1.58 | (-7.21, 4.02) | 6.29 | (-0.23, 12.61) |
| **Years of stay** |  |  |  |  |  |  |  |  |
| Below 5 years | Ref | Ref | Ref | Ref | Ref | Ref | Ref | Ref |
| 6-10 years | 1.95 | (-1.55, 5.44) | 1.27 | (-1.91, 4.45) | 1.04 | (-0.60, 2.69) | 1.37 | (-0.29, 3.02) |
| Above 40 yrs | 1.94 | (-1.29, 5.17) | 1.13 | (-1.84, 4.09) | 0.49 | (-1.06, 2.04) | 0.98 | (-0.54, 2.51) |
| **Occupation** |  |  |  |  |  |  |  |  |
| Unemployed | Ref | Ref | Ref | Ref | Ref | Ref | Ref | Ref |
| Trading | -7.94* | (-11.24, -4.64) | -5.23* | (-8.59, -1.87) | 0.78 | (-5.) | -2.36* | (-4.31, -0.41) |
| Farmer | 1.57 | (-2.45, 5.59) | 2.91 | (-1.15, 6.97) | -1.59 | (-3.65, 0.48) | -0.65 | (-3.00, 1.69) |
| Formal sector | -3.52 | (-18.88, 11.84) | -8.87 | (-24.20, 6.46) | -8.55* | (-12.96, -4.15) | -9.87* | (-15.41, -4.32) |
| Artisan | -7.00* | (-11.41, -2.61) | -6.22* | (-10.48, -1.96) | -6.57* | (-9.29, -3.86) | -5.30* | (-8.15, -2.45) |
| Others | -10.81* | (-15.62, -5.99) | -12.19* | (-16.85, -7.51) | -1.25 | (-5.89, 3.39) | -2.04 | (-6.77, 2.69) |
| **Heard of FGS** |  |  |  |  |  |  |  |  |
| No | Ref | Ref | Ref | Ref | Ref | Ref | Ref | Ref |
| Yes | -9.27* | (-12.58, -5.97) | -6.99* | (-10.15, -3.84) | 0.77 | (-1.07, 2.62) | -0.16 | (-1.86, 1.54) |

* Statistically significant (p-value <0.05), Coeff= regression coefficient, CI= Confidence interval at 95%

Table 3: A regression analysis of women background characteristics and practices towards FGS in the Lower Manya-Krobo Municipal and the Shai Osudoku District

| Variable s | **Shai Osudoku district (Case district)** | | | | **Lower Manya-Krobo municipal (Control** | | | |
| --- | --- | --- | --- | --- | --- | --- | --- | --- |
|  | **Bivariate (Crude)** | | **Multivariate (Adjusted)** | | **Bivariate (Crude)** | | **Multivariate (Adjusted)** | |
|  | **Coeff.** | **CI** | **Coeff.** | **CI** | **Coeff.** | **CI** | **Coeff.** | **CI** |
| **Educational** |  |  |  |  |  |  |  |  |
| None | Ref | Ref | Ref | Ref | Ref | Ref | Ref | Ref |
| Primary | -5.14* | (-10.10, -0.19) | -3.48 | (-8.32, 1.35) | -5.42* | (-10.02, 0.82) | -4.16* | (-8.32, -0.00) |
| Secondary | -11.95* | (-17.28, -6.62) | -7.61* | (-13.02, -2.19) | -11.04* | (-16.11, -5.97) | -7.92* | (-12.84, -3.01) |
| Tertiary | -22.74* | (-42.53, -2.95) | -20.57* | (-41.02, -0.12) | -16.0* | (-29.63, -2.63) | -10.62 | (-26.37, 5.14) |
| **Years of stay** |  |  |  |  |  |  |  |  |
| Below 5 years | Ref | Ref | Ref | Ref | Ref | Ref | Ref | Ref |
| 6-10 years | 6.08* | (1.78, 10.39) | 4.09 | (-0.06, 8.24) | 13.33* | (9.16, 17.51) | 8.31* | (4.19, 12.43) |
| Above 10 years | 5.20* | (1.21, 9.19) | 4.59* | (0.78, 8.42) | 13.83* | (9.91, 17.75) | 12.11* | (8.34, 15.88) |
| **Occupation** |  |  |  |  |  |  |  |  |
| Unemployed | Ref | Ref | Ref | Ref | Ref | Ref | Ref | Ref |
| Trading | -1.76 | (-5.88, 2.35) | -2.77 | (-6.89, 1.34) | -0.41 | (-463, 3.81) | -3.35 | (-7.69, 0.98) |
| Farmer | 9.14* | (4.11, 14.17) | 7.41* | (2.33, 12.49) | 18.34* | (12.96, 23.71) | 14.03* | (8..47, 19.59) |
| Formal sector | -10.0 | (-29.18, 9.18) | -3.97 | (-23.99, 16.05) | -7.25 | (-18.71, 4.21) | -5.63 | (-19.29, 8.03) |
| Artisan | -2.08 | (-7.57, 3.42) | -2.27 | (-7.71, 3.17) | 1.72 | (-5.35, 8.78) | -0.86 | (-7.62, 5.90) |
| Others | -11.95* | (-17.96, -5.94) | -10.74* | (-16.80, -4.67) | -7.25 | (-19.33, 4.83) | 0.07 | (-11.68, 11.82) |
| **Heard of FGS** |  |  |  |  |  |  |  |  |
| No | Ref | Ref | Ref | Ref | Ref | Ref | Ref | Ref |
| Yes | -0.13 | (-4.38, 4.12) | 2.07 | (-2.02, 6.15) | -4.74* | (-9.25, -0,23) | -0.63 | (-4.88, 3.62) |

*Statistically significant (p-value <0.05), Coeff= regression coefficient, CI= Confidence interval at 95%
